# Integrating population-level effects into the regulatory assessment of endocrine disrupting substances

**DOI:** 10.1093/inteam/vjae039

**Published:** 2025-01-06

**Authors:** Charles R E Hazlerigg, Alice Tagliati, Valery E Forbes, Andre Gergs, Nina Hallmark, Lorraine Maltby, Lennart Weltje, James R Wheeler

**Affiliations:** Enviresearch Ltd, Newcastle-upon-Tyne, United Kingdom; School of Natural and Environmental Sciences, Newcastle University, Newcastle-upon-Tyne, United Kingdom; Enviresearch Ltd, Newcastle-upon-Tyne, United Kingdom; Department of Biological Sciences, Florida Atlantic University, Boca Raton, FL, United States; Bayer AG, Monheim, Germany; Bayer AG, Monheim, Germany; School of Biosciences, University of Sheffield, Sheffield, United Kingdom; BASF SE, Agricultural Solutions—Ecotoxicology, Limburgerhof, Germany; Division of Plant Pathology and Plant Protection, Georg-August-University Göttingen, Göttingen, Germany; Corteva Agriscience, Bergen op Zoom, The Netherlands

**Keywords:** hazard assessment, chemical regulations, population modeling, vertebrate populations, endocrine disruptors

## Abstract

Population modeling, field studies, and monitoring approaches have all been proposed for assessing the relevance of adverse effects of endocrine disrupting chemicals (EDCs) at the population level for nontarget (wild) vertebrates, but how these approaches should be used in the regulatory hazard assessment is unclear and not detailed in the relevant European Guidance Document. A literature review focused on identifying published approaches assessing the population relevance of adverse effects from EDCs was performed, and, subsequently, 47 primary research papers were evaluated. By extracting from these sources, a novel approach was developed with guiding principles for assessing adverse effects of EDCs at the population level considering (i) choice of focal species, scenarios (and models), (ii) the individual level apical endpoints to be considered, (iii) the magnitude of effect to be imposed, (iv) for what duration effects should be imposed, (v) whether individuals repairing the damage from exposure should be included, (vi) the population-level endpoints to be considered, and (vii) what threshold to set for defining an adverse effect at this level. Recommendations for modeling and field and monitoring studies are included. Case studies are also presented to demonstrate how the proposed approach might be implemented. Although some aspects (e.g., choice of focal species, model/experimental scenario, monitoring study assessment) require further consideration, this should not prevent the use of this approach in a regulatory EDC assessment context. As such, we propose that the approach be used immediately to implement population modeling and perform field studies within this regulatory context. We envisage that consistent application of these principles will encourage regulatory developments in this critical area to provide a much needed level of clarity in the EDC assessment for all stakeholders.

## Teaser

- The majority of the literature reviewed for population relevance assessment of endocrine disrupting chemicals (EDCs) used modeling approaches and applied them to fish (rather than field or monitoring studies, applied to terrestrial vertebrates).
- The approaches to population relevance assessment of EDCs in the literature were diverse, highlighting the need for a consistent method for application in a regulatory context.
- We provide an approach to perform a population relevance assessment of EDCs that may be used in a regulatory context, identifying seven key points that need to be addressed in this process.
- We consider our approach against four case studies from the literature to provide an illustration of how such a population relevance assessment for EDCs may be performed.

## Introduction

The potential impact of endocrine disrupting chemicals (EDCs) to wildlife has been a concern for many years (e.g., WHO, 2002). In the European Union (EU), Regulations (EU) No. 2017/2100 (EC, 2017) and (EU) No. 2018/605 (EC, 2018) and the accompanying Guidance Document by the European Chemical Agency (ECHA) and European Food Safety Authority (EFSA; ECHA & EFSA, 2018) define the scientific criteria for identifying EDCs for biocidal product (BP) and plant protection product (PPP) active substances. A chemical substance is considered an endocrine disruptor if it causes an adverse effect, it has endocrine activity, and there is a biologically plausible link between the adverse effect and the endocrine activity. These criteria apply unless nonhuman relevance or nonpopulation relevance is demonstrated for human health or the environment, respectively ([EU] No. 2017/2100 [EC, 2017]; [EU] No. 2018/605 [EC, 2018]). These criteria do not support a risk assessment approach, but instead require a hazard only-based method as dose/exposure is not considered. Other geographies also require that substances are evaluated for their endocrine disrupting properties as part of the registration process, but a risk-based approach is used (See online supplementary material). Although hazard- and risk-based EDC assessments have long been debated (e.g., Wheeler et al., 2012), the aim of both approaches is the protection of wildlife populations with differing levels of conservatism to cover actual and perceived uncertainties. In this article, the scope is aligned with the EU regulatory context for the environment and will only consider vertebrates (terrestrial and aquatic) and the estrogen, androgen, thyroid, and steroidogenic (EATS) modalities in the ecotoxicological hazard assessment.

The EU hazard-only method is mostly well defined for human health to meet the protection goal at the individual level. By contrast, the methodology for the determination of whether the protection goal of population relevance for wild vertebrates has been demonstrated is currently insufficient. For wildlife, generally known as nontarget organisms (NTOs), the regulatory protection goal is the maintenance of their populations. According to the assessment strategy proposed by the ECHA & EFSA (2018) guidance, any measured endocrine-mediated adverse effect on growth, development, or reproduction from laboratory studies is conservatively considered relevant at the population level, unless there is evidence demonstrating the contrary. This is in principle performed by considering the adverse outcome pathway (AOP) concept within a weight-of-evidence (WoE) approach. An AOP describes a mechanistic connection of events linking a toxicant-induced molecular initiating event (MIE) to an adverse outcome (Ankley et al., 2010). However, it is inaccurate to conclude that an MIE automatically results in an adverse outcome leading to a population change (Lagadic et al., 2020), or that the effects occurring at the population level are necessarily proportional to the effects measured on individual organisms (Forbes et al., 2016), as assumed by ECHA & EFSA (2018) in the absence of additional data. Providing such resource-intensive additional evidence is not routinely required for the assessment of any chemical substance (conservative conclusions are drawn from laboratory data). However, when there is a significant need to determine whether safety concerns can be adequately addressed, more advanced approaches to assess the relevance of laboratory-measured endocrine adverse effects to the population could be explored. Population modeling and field and monitoring studies are considered as potential sources of such evidence; however, no guidance on how to perform or interpret such studies is provided. To support the development of workable guidance to address these gaps, the European Centre for Ecotoxicology and Toxicology of Chemicals (ECETOC) initiated an expert group to scope guiding principles to determine the potential population relevance of endocrine-mediated adverse effects for nontarget vertebrate organisms.

The objective of this article is to suggest a process to practically assess the adverse population-level endocrine-disrupting effects of chemical substances using population models and field and monitoring studies within a hazard assessment. Previous work by Crane et al. (2019) started this for population models by developing a conceptual overview of how the process may be implemented. We take this further, not only by considering field studies and monitoring studies in line with the guidance, but also very specifically defining some key points regarding implementation necessary to routinely use the approach in a regulatory context. Case studies to explore these approaches are also described. This study is based on current experiences with PPPs and BPs in the EU, where the regulatory requirements for assessment of endocrine properties are already established. However, the scientific principles of assessing population relevance of EDCs are considered broadly relevant to all chemicals.

## Methods

A narrative literature review was conducted to identify sources exploring the population-level responses of vertebrates to exposure to EDCs in Web of Science (https://www.webofscience.com) using broad-ranging keywords associated with EDCs and population-level responses (See details in the online supplementary material). This search string yielded 1,127 retrievals (after duplicate removal) on November 30, 2022. Articles were manually screened based on evaluation of title and abstract, with no limit on publication date, and fulfilled the following criteria that were defined a priori:

- Stressor: chemical exposure
- Mechanism: endocrine disruption, any modality
- Effect: population level, any attribute
- Taxa: fish, mammal, amphibian, bird

These selection criteria were deliberately broad, as this is an emerging research area and a limited number of suitable articles were expected. Four further articles not originally captured in the literature search, but known by the authors to be relevant, were added to this list. Three did not use any of the EDC terms in the search string: One generally concerned ecological risk assessment but was also relevant to endocrine disruption (Raimondo et al., 2018), a second discussed pesticide-induced chronic effects that turned out to be a hypothetical endocrine active chemical (EAC) in the main body of the text (Dalkvist et al., 2009), whereas the third concerned a specific mechanism-of-action at the molecular level without using the more general EDC terms (Conolly et al., 2017). The fourth article was not yet published at the time of the search (Hazlerigg et al., 2023). In total, 47 articles were identified, which were used to develop the principles presented in the next section and four were used as case studies (see *Applying the approach: case studies* section and the online supplementary material). Fish were the most studied taxa, accounting for 34/47 (72%) of sources. Modeling approaches were most common, accounting for 29/47 (61%) of sources.

## Development of an approach for population relevance assessment

The review of the literature identified several critical aspects that need to be addressed to assess the population relevance of an EDC, irrespective of whether a modeling, monitoring, or field study approach would be undertaken. We identify these as the following seven points: (i) focal species and ecological scenario, (ii) individual-level apical endpoints, (iii) magnitude of effect, (iv) duration of effect, (v) damage repair, (vi) population-level endpoints, and (vii) threshold for population response. Consideration of these key points may be used to design and evaluate an appropriate modeling, monitoring, or field study for any chemical to determine the population relevance of any adverse effects observed in the laboratory. This method has been developed with a view to operationalizing a population relevance assessment in line with the EU regulatory framework. However, potential chemical-, species- and use-specific refinements are also explored briefly but are not currently included in our recommendations as they require further development. In the next sections, each of these seven points will be presented in detail.

### Focal species and ecological scenario

The literature reviewed often used standard laboratory species (e.g., fathead minnow [*Pimephales promelas*], Miller et al., 2022) or focal species commonly used in chemical assessment (e.g., field vole [*Microtus agrestis*], Dalkvist et al., 2009), although species not commonly used in chemical assessment also featured to a lesser extent (e.g., perch [*Perca fluviatilis*], Brown et al., 2005). The ecological scenarios used also varied widely in spatial scale (e.g., small pond [20 m^2^] and terrestrial enclosures [0.5 ha] to large rivers and terrestrial landscapes [10 km^2^]), temporal scale (e.g., a few weeks to 120 years) and ecological community (e.g., single species or full ecosystem). It is well known that species may differ in their sensitivity to EDC exposure (e.g., Werner et al., 2006) and even within a species, different life-stages may differ in their sensitivity (e.g., Marty et al., 2022), so sensitive life-stages should be included within any population-level assessment. Meanwhile, population-level processes (e.g., density dependence, competition) and variability in life history prevent simple extrapolation of toxicological sensitivity to the population level, i.e., species that are sensitive at the individual level are not necessarily the species most sensitive at the population level (e.g., Awkerman & Raimondo, 2018). As such, a wide range of potential species might be selected for use in an EDC assessment and so the concept of focal species used in PPP risk assessment (e.g., birds and mammals; EFSA, 2023) may also be applied here. The critical aspect is that a relevant vulnerable species is selected as the focal species to use in the assessment, thereby covering the potential for harm to other species. Ibrahim et al. (2014) performed such an analysis for 23 European fish species and concluded (using matrix population models) that Eurasian minnow (*Phoxinus phoxinus*) was least resilient to effects on fertility, brook lamprey (*Lampetra planeri*) was least resilient to effects on juvenile mortality, and pike (*Esox lucius*) was least resilient to effects on adult mortality. As such, no single species may be considered most vulnerable to all possible effects. Meanwhile, EFSA (2023) also proposes a wide range of potential focal species for mammals and birds depending on the proposed use of the product. Whether the focal species used in standard risk assessments are appropriate for use in a hazard-based EDC assessment is currently unresolved. Therefore, at the current time, a practical solution is to use species that may be considered vulnerable due to the combination of their toxicological and ecological sensitivity (i.e., they should fall on the left-hand side of a species sensitivity distribution and their populations are known to have a high degree of sensitivity (low resistance) to changes in the individual-level effects being imposed).

### Individual-level apical endpoint

A range of endpoints were considered in the reviewed literature: from suborganismal biomarkers (e.g., vitellogenin, Burkhardt-Holm et al., 2008), to apical endpoints such as development (e.g., Awkerman & Raimondo, 2018) and behavior (e.g., Mintram et al., 2018). The OECD Conceptual Framework for Testing and Assessment of Endocrine Disrupting Chemicals presents the standardized test guidelines for evaluating chemicals for endocrine activity/disruption and how to interpret the results (OECD, 2018) consistent with the regulatory guidelines (ECHA & EFSA, 2018). To assess population relevance of an EDC, it is essential that an adverse effect on an apical endpoint (e.g., fecundity) has been demonstrated and quantified at the individual level as a result of an endocrine mechanism. Effects at lower levels of biological organization (i.e., an effect on a mechanistic parameter) should also be reflected in an effect on an apical endpoint to be consistent with the AOP concept and therefore justify investigation at the population level. The apical endpoints associated with these standardized studies are summarized in Marty et al. (2017) and Crane et al. (2019). These are the endpoints that may be selected for investigation using the approach proposed here. Although seemingly simple, care must be taken to ensure specific endocrine effects are disentangled from those of generalized toxicity (i.e., responses that affect the whole body) as well as primary versus secondary effects, which may result in inaccurate data interpretation (Marty et al., 2018). If multiple endocrine-mediated effects are observed for a given substance, then all should be included within the population level assessment.

### Magnitude of effect

Many of the modeling studies identified in the literature review used a dose-response relationship (e.g., Miller et al., 2022), where the magnitude of effect depended on the concentration of the chemical. Although scientifically robust and justifiable, this is inconsistent with the need for a hazard assessment. In the reviewed field studies, a single concentration of the EDC was used, with that concentration or dose informed by the results of monitoring studies (e.g., Kidd et al., 2007) or from a representative use (e.g., Caslin & Wolff, 1999). As the exposure level chosen was not selected to specify only adverse effects mediated by an endocrine mode of action, these may not be relevant for endocrine hazard assessment within the current regulatory construct. Finally, as the monitoring studies relied on concentrations (and effects) present in the environment (e.g., Ussery et al., 2021), these are also inconsistent with the needs of an assessment of only endocrine-mediated effects within a hazard context.

One recent modeling study isolated the magnitude of effect to only that resulting from an endocrine mode of action by using the magnitude of effect observed at the maximum tolerated concentration (MTC) in chronic laboratory fish studies (Hazlerigg et al., 2023). The MTC (and the analogous maximum tolerated dose, MTD) is the threshold of generalized toxicity, below which the tested concentrations (or doses) may induce measurable effects on the organisms but are not too high to induce generalized toxicity (Hutchinson et al., 2009) and confound endocrine effect interpretation. Data packages may be extensive, including multiple studies with multiple endpoints, each with their own MTC/MTD and magnitude of effect. The magnitude of effect should be endpoint-specific and taken from the highest tier test available (i.e., highest level of the OECD Conceptual Framework), as these studies integrate all potential individual adverse effects and so represent the worst case from the toxicological perspective. Although there are no examples of field studies using the MTC approach, if a test concentration could be determined that resulted in an individual-level adverse effect equivalent to that observed in laboratory studies (at the MTC) and this exposure and effect on individuals could be accurately implemented in the field, then it would be theoretically possible to perform an empirical study for a hazard assessment. It should be noted that regulatory approval to conduct these types of manipulation field studies (i.e., vertebrate species with the expectation of severe effects) may face serious hurdles (e.g., due to a commitment to replacement, reduction, and refinement of animal testing in Europe). As such, population modeling may be the preferred approach for an EDC assessment as it is easier to implement effects associated with the MTC (including options to explore different scenarios), and avoid the technical and ethical challenges inherent in a comparable field study. As endocrine-mediated effects associated with an MTC are commonly observed at concentrations lower than those effects mediated by other modes of action, the need to consider any other nonendocrine-mediated effects in the population relevance assessment will often be unnecessary. However, on occasion, a substance may have both endocrine-mediated and nonendocrine-mediated effects at similar test concentrations, in which case, scientifically speaking, all effects would be expected to occur simultaneously in wild populations. Should this be the case, the nonendocrine-mediated effects may also be included in the population relevance assessment; however, they should be included according to a risk assessment paradigm (i.e., on the basis of exposure) and not according to the hazard-based approach being recommended for endocrine-mediated effects in this study. This dichotomy is a direct result of the different regulatory criteria applied to endocrine- and nonendocrine-mediated effects and highlights the challenges we are attempting to deal with in making recommendations regarding how to perform a hazard-based population-level assessment.

### Duration of effect

A wide range of durations of effect, from 10 days to 200 years (where effects are also imposed on subsequent generations), were observed in the results of the literature review (e.g., Brown et al., 2005; Hazlerigg et al., 2014; Li et al., 2020). Furthermore, for some studies, effects were imposed continuously (e.g., Mintram et al., 2018) whereas in others, they were intermittent (e.g., Li et al., 2020). In another, the duration of effects was dependent on the FOrum for Co-ordination of pesticide fate models and their USe (FOCUS) exposure profiles from the representative use (Hazlerigg et al., 2023). What these studies show is that the duration of effects has a significant impact on the population response. For example, imposing changes on sex ratio in zebrafish for one year or 10 years led to a far greater reduction in population abundance and biomass in the latter (Hazlerigg et al., 2014). If we consider EU Regulations (EU) No. 2018/605 (EC, 2018) and (EU) No. 2017/2100 (EC, 2017), then continuous effects (not intermittent effects) should be imposed, as this is a hazard assessment. As such, one method would be to impose the magnitude of effect on an endpoint for a continuous period of one year. One year is selected as a reasonable worst-case approach, as generally (i) many chemicals should have declined significantly within a year, given the restrictions for authorization required by other hazard requirements such as “Persistence” in the Persistence, Bioaccumulative and Toxic (PBT) classification (e.g., [EC] No. 1107/2009; European Parliament and Council, 2009); (ii) this considers the likely change in land use each year due to common crop rotation strategies (although not for perennial crops like vineyards); and (iii) this is consistent with the breeding cycle for many European vertebrate wildlife of interest to this assessment. In cases where these assumptions cannot be justified for the proposed use, a longer duration may be more suitable.

### Damage repair

The reviewed literature predominantly included examples where damage repair at the individual level was possible. In many cases, this was a direct result of using a dose-response to impose EDC effects on individuals (i.e., when the exposure decreased, damage repair was possible). Some modeling examples also considered scenarios without damage repair with the use of long-term continuous exposure (e.g., Mintram et al., 2018). Whether individuals affected by an EDC repair the damage or not once the EDC is no longer present is immaterial for the approaches we described above where effects are imposed continuously (as population recovery cannot be considered from a regulatory standpoint). For some approaches suggested as potential refinements (see *Possible refinement considerations* section), this needs to be considered due to the intermittent nature of the imposed effect(s). Given the range of different endpoints that might be affected by EDCs, it is difficult to prescribe one approach for all. For example, decreased growth following exposure to the estrogen ethynylestradiol (EE2) during developmental life-stages in zebrafish is reversible (Baumann et al., 2014) whereas effects on other endpoints are not. Whether individual repair for a given endpoint can be generalized across different taxa a priori needs further research. In the meantime, whether an effect should be recoverable/reversible at the individual level should be considered using knowledge of the mode of action.

### Population-level endpoints

Population abundance (total and recruited), biomass (total and recruited), and size distribution (e.g., ratio of highest and lowest size classes by West et al., 2006) were generally common in the literature, whereas population growth rate was specifically common for matrix modeling (e.g., Gleason & Nacci, 2001), which can be a useful measure of relative vulnerability as well as recovery potential. Meanwhile, others, such as population “energy storage” (defined as cumulative condition index, West et al., 2006), were less common. Although this latter metric is interesting and potentially informative, its use may be limited to certain situations (e.g., energy-budget modeling). Population abundance, biomass, and size distribution are considered appropriate for the proposed use, consistent with the recommendations by Crane et al. (2019).

### Threshold for population response

The thresholds used to define whether an adverse effect had been observed in the population ranged from a 5% (Mintram et al., 2020) to 15% (Mintram et al., 2018) deviation from the control in modeling, to outside the normal operating range ± 2 *SDs* in monitoring (Ussery et al., 2021) and statistically significant results in field studies (Caslin & Wolff, 1999). Although the use of standard statistical tests to determine adverse population responses in field studies is appropriate, this is not the case for population models where it is simple to increase replication by performing more simulations and therefore change the value for statistical significance. As such, the recent methods proposed by EFSA (2023) are more appropriate than those presented in the literature. EFSA (2023) states that the following two criteria should be used for assessing whether an adverse population-level effect has been observed: (i) the mean of the exposed population falls below the lower 95^th^ percentile of the control and (ii) the lower 95^th^ percentile of the exposed population is consistently lower than the lower 95^th^ percentile of the control population. This method will give a consistent outcome, regardless of whether more model simulations are performed. The use of the EFSA (2023) criteria and the concept of a normal operating range considers ecology and the actual dynamics of the population.

Relatedly, population recovery may be easily studied, with the removal of the EDC from the model/study area. However, it can be deduced from EU Regulations (EC) No. 1107/2009 (European Parliament and Council, 2009) and (EC) No. 528/2012 (European Parliament and Council, 2012) that population recovery will not be considered, as once an adverse population effect has been observed, the chemical will be identified as an EDC, irrespective of whether the population recovers once exposure to that chemical is stopped. This is a worst-case scenario consistent with the hazard assessment requirements; hence population recovery is not considered further here.

A summary of the considerations and recommendations for applying the proposed approach is given in Table 1.

**Table 1.** Proposed approach: considerations and recommendations for the development of modeling, monitoring, and field studies for determining the population relevance of EDCs.

Element	Population modeling	Field study	Monitoring
**Focal species and ecological scenario**	Identify suitable, vulnerable focal species and appropriate ecological scenarios		

**Individual-level endpoint(s)**	Apical endpoints from Marty et al. (2017), ECHA & EFSA (2018) and Crane et al. (2019)		

**Magnitude of effect**	Associated with chronic MTD or MTC*		Associated with environmentally relevant concentration (note: this is inconsistent with the EDC EU regulation)

**Duration of effect (on individuals)**	Continuous for one year		Continuous, associated with environmental concentration (note: this is inconsistent with the EDC EU regulation)

**Damage repair (in individuals)**	No		Associated with exposure profile and species physiology

**Population-level endpoint(s)**	Abundance, biomass, size distribution		

**Threshold for population response**	EFSA 2023 criteria, Normal Operating Range	Statistical significance (assuming sufficient study power)	Statistical significance/Reference site

*Note.* MTD = maximum tolerated dose; MTC = maximum tolerated concentration; EDC = endocrine disrupting chemical.

* Or maximum solubility/10 mg/L (regulatory threshold for chronic testing) if MTD/MTC is not reached. Must be a primary effect and not the result of a secondary toxic effect.

### Possible refinement considerations

If the initial population relevance assessment is positive (i.e., an adverse effect on the population is demonstrated) then further modelling refinement may be performed. These suggestions are presented more loosely as an incomplete list of points for further discussion. In general, chemical-, species-, and use-specific refinements using greater realism but also requiring greater effort (i.e., model parameterization, simulation, and analysis) may be considered.

Initial population assessment assumes that the MTD/MTC has been clearly defined for each study and that the AOP is clearly understood for the endocrine mode-of-action. Even with thoughtful test design, only certain concentrations are tested in laboratory studies, so the MTD/MTC is limited to one of those and the amount of supporting mechanistic information (e.g., biomarkers) available to differentiate between endocrine-mediated and generalized toxicity can vary. Often AOPs may be complex and need to be further investigated resulting in a potential refinement of type of effect and its magnitude.

The one-year duration of effect in the initial population assessment was selected as a reasonable worst-case duration. However, in an assessment only focused on endocrine-mediated effects on fecundity for example, consideration of a shorter duration of effect informed by the sensitive period and breeding season of the selected species (e.g., three months for stickleback) could be appropriate.

EU Regulation (EC) No. 1107/2009 (European Parliament and Council, 2009) includes the derogation in the nonauthorization of an active substance identified as an EDC if exposure is negligible. If the term “negligible” can be defined, and the threshold value for negligible quantified, then exposure below that threshold would result in no effects on individuals being imposed in the model. This was an approach explored by Hazlerigg et al. (2023) and Dalkvist et al. (2009). However, in both these studies, negligible exposure is quantified based on an effects endpoint and a safety factor. Although the safety factor was more protective (greater) than that used in standard risk assessment, the concept is still risk-based and not hazard-based, seemingly incompatible with the EDC identification guidance (ECHA & EFSA, 2018). Then again, a recent literature review of the term “negligible exposure” in relation to endocrine substances concluded that where information on mode of action, validated test systems, and regulatory acceptable concentrations are available, and low-dose effects with nonmonotonic dose-response relationships are excluded, then the exposure is understood to be negligible if demonstrated to be below toxicologically relevant thresholds for the representative uses (UBA, 2023). Given this conclusion, it appears that the refinement presented in Hazlerigg et al. (2023) and Dalkvist et al. (2009) may be supported if this additional information is available and meets this recently suggested definition.

We also consider all individuals from a population to be affected in the initial population assessment during the effects period. In aquatic systems, we might conservatively consider full mixing of a chemical in the environment so all organisms may be affected, but in terrestrial systems, this would be dependent on many factors, such as movement of the organisms, consumption of residues, and use of the product, among others.

These possible refinements might not be limited to the implementation of effects but also consider the evaluation criteria for a population response. The threshold for population effects is calculated in the initial population assessment using the EFSA (2023) criteria. This is the regulatory standard presently; however, developments in this area are moving rapidly at the current time. For example, it is debateable whether strict application of criterion 2, where a population response will be recorded if the lower 95th percentile is “…consistently lower than that of control… (…on a limited number of isolated timepoints only.)” (EFSA, 2023, p. 107), is appropriate. As such, alternate methods for assessing model outputs may be considered with justification.

## Applying the approach: case studies

None of the studies identified by the literature review fully comply with the approach proposed in this article. Therefore, one example from the literature that most closely matched the approach recommended in this article is presented in the following section (*Mammalian modeling*), with a further three examples provided in the online supplementary material (one fish modeling, one fish field study, and the third a mammalian field study; all four examples are summarized in Table 2). A brief summary of each case study is provided, detailing how each of the seven points has been addressed, and finally, we review this against the guidance and recommendations within this manuscript for use in a regulatory context. There are several good monitoring studies available for EDCs (e.g., the *Fischnetz* project; Burkhardt-Holm et al., 2008; Ussery et al., 2021), which may be useful in a WoE approach for the renewal of an active substance. However, it is currently difficult to see how these could be used effectively in a prospective hazard assessment as required by Regulation (EU) No. 2018/605 (EC, 2018) and as such, no monitoring case studies have been provided.

**Table 2.** A summary of case studies and their compliance with the proposed approach to assess the population relevance of endocrine disrupting chemicals.

Element	Study			
	Fish modeling	Mammal modeling (Refinement)	Fish field study	Mammal (semi) field study
	Conolly et al. (2017)	Dalkvist et al. (2009)	Kidd et al. (2007)	Caslin & Wolff (1999)
**Focal species and ecological scenario**	Fathead minnow	Field vole	Pearl dace, fathead minnow, lake trout, white sucker	Gray-tailed vole
**Individual-level endpoint(s)**	Fecundity	Male sterility and mating success	Length, weight, histology, biomarkers (vitellogenin, sex steroids), (fertilization rate, hatch success, deformation rate*)	Recruits/female, Sex ratio, Biomarkers (testes size, plasma testosterone, hip glands)
**Magnitude of effect**	*From concentration-response test*	*Hypothetical*	*Based on concentrations observed in monitoring data*	*Based on concentrations from use of representative product*
**Duration of effect (on individuals)**	Ten years^^^	*Thirty year exposure profile from proposed use and threshold for effect (25 mg/kg bw/d)*	Three years^^^	*Two months*
**Damage repair (in individuals)**	No	No	Determined by natural processes	Determined by natural processes
**Population-level endpoint(s)**	Abundance (Proportion of carrying capacity)	Abundance	Abundance and body size distribution	Abundance
**Threshold for population response**	*20% deviation from control*	*Not specified*	Statistical testing	Statistical testing

*Note.* Cells with text in normal font are consistent with the proposed approach, whereas those with text in italics are not. The mammalian modeling case study is discussed in the article, and details of the other three case studies may be found in the online supplementary material.

* These additional endpoints were investigated in complementary laboratory testing in Werner et al. (2006).

^ These case studies used durations longer than the one year proposed so are considered acceptable as more worst-case.

### Case study: mammalian modeling

#### Study description

Dalkvist et al. (2009) used a field vole (*Microtus agrestis*) individual based model (IBM) with an ALMaSS model simulating a 10 × 10 km^2^ Danish landscape to assess the effects of a hypothetical EDC on vole populations. An initial 30-year period was modeled without chemical exposure, followed by 30 years of continuous exposure, and finally, 60 years of no exposure to assess population recovery. The exposure profile was based on an annual application rate of 750 g/ha applied directly to the surface vegetation, with spray drift to off-crop areas included in the modeled landscape. Residue decline based on the chemical’s half-life was then used to define the temporal exposure profile. For each timestep (daily), any pregnant dams at 16–21 days gestation exposed to residues (in their diet) above the laboratory determined no observed effect level (NOEL) of 25 mg/kg bw/day were considered exposed to the EDC. Fifty percent of the male offspring of any exposed dam were sterile and could not contribute to reproduction for their lifespan. Although theoretical, these effects were loosely based on those observed for the fungicide vinclozolin and assumed to be endocrine-mediated for the purpose of the study. The population abundance was recorded at every timestep and compared with results from control simulations without chemical exposure and effects. A maximum reduction in population abundance of just over 8% was observed in the exposed scenarios compared with the unexposed control, though the authors did not state a threshold for what was considered an adverse population-level effect.

#### Implementation of the seven points of the proposed approach

The field vole is a commonly used focal species in assessing the risk to mammals from PPPs (EFSA, 2009, 2023). A small herbivore with a dietary preference for grasses and sedges, it is most commonly found in rough grassland and pasture (Gurney et al., 1998). The low body weight and high feeding rate of the field vole make it susceptible to an EDC. The model structure (IBM) and scenario (agricultural land use in Denmark) are appropriate.

The chosen individual-level endpoint (sterility) has a direct bearing on reproductive success (described as relevant by Marty et al., 2017) and is therefore of interest, given the likely endocrine mode of action. The magnitude of effect used (50%) was hypothetical, not based on empirical laboratory data. The duration of effect emerged from the combination of exposure profile (based on representative use of a product and decline in the environment) and the use of a threshold for effects (NOEL); this is an example of a refinement. Dams that were exposed did show damage repair and could produce healthy males in the future, whereas 50% of unborn males exposed to a dose above the threshold remained sterile for their entire lives.

The population response is reported as changes in daily abundance within the landscape, although the threshold for significance is not clearly defined (except for population recovery, which is defined as the time taken for the exposed population abundance to exceed that in the control simulation).

#### Conclusions for regulatory use

Although this case study implements some of the recommendations within this article, further work would be required to use this in a regulatory context. First, establishing species vulnerability is necessary to identify the appropriate focal species and requires a species to be sensitive to the toxicity of a chemical and present in the area of chemical application and determining the resilience of the population once exposed. Although the field vole may be appropriate for grassland scenarios, modeling other species may be more appropriate when considering other arable crops. For example, Liu et al. (2013) present an IBM for the wood mouse (*Apodemus sylvaticus*) that may be suitable for agricultural landscapes where seed treatments are common, due to their omnivorous diet. Furthermore, the cyclic “boom and bust” population dynamics of voles suggest a high compensatory reserve at the population level, meaning population resilience may be larger compared with other species. It is not clear whether considering larger herbivores may be more appropriate when performing an EDC population relevance assessment. A study into the mammalian equivalent of the fish community presented by Ibrahim et al. (2014) would be beneficial in clarifying suitable focal species that may be considered potentially vulnerable to EDCs due to specific life-history characteristics.

Second, assuming that sterility is the most sensitive endpoint and there are no other adverse effects observed at the individual level for the substance being assessed, a suitable laboratory study should be available from which an MTD can be determined, and the level of sterility observed at that concentration should then be imposed in any modeling. The restriction of effects to only pregnant dams exposed during days 16–21 gestation (and not during other gestational time windows) would also need to be clearly reasoned based on laboratory data.

Third, the combination of exposure profile and effects threshold (NOEL) is a higher tier refinement and this is unlikely to be accepted until the threshold for what constitutes “negligible exposure” is agreed. As such, modeling without this refinement should also be provided, where effects are imposed for a set period of time (one year) on all field voles within the simulations regardless of exposure.

Finally, the threshold for population relevance defined by EFSA (2023) could be applied to the model outputs in this case study. Beyond the initial model simulations (with a default NOEL of 25 mg/kg bw/day), the authors also presented further modeling using a range of other NOELs (50–1.56 mg/kg bw/day). This additional modeling is equivalent to an exposure multiplication factor of 0.5–16. Although the additional simulations were not consistent with the recommendations presented here, it does illustrate how the concept of negligible exposure might be implemented in a higher tier refinement. Interestingly, the highest multiplication factor would have resulted in a population decline of approximately 15% and changed the outcome of the hazard assessment regarding population relevance (if using the 10% change as the threshold for significance). Population recovery need not be assessed, as it is not considered acceptable in a regulatory context.

## Conclusion

**Figure 1.**
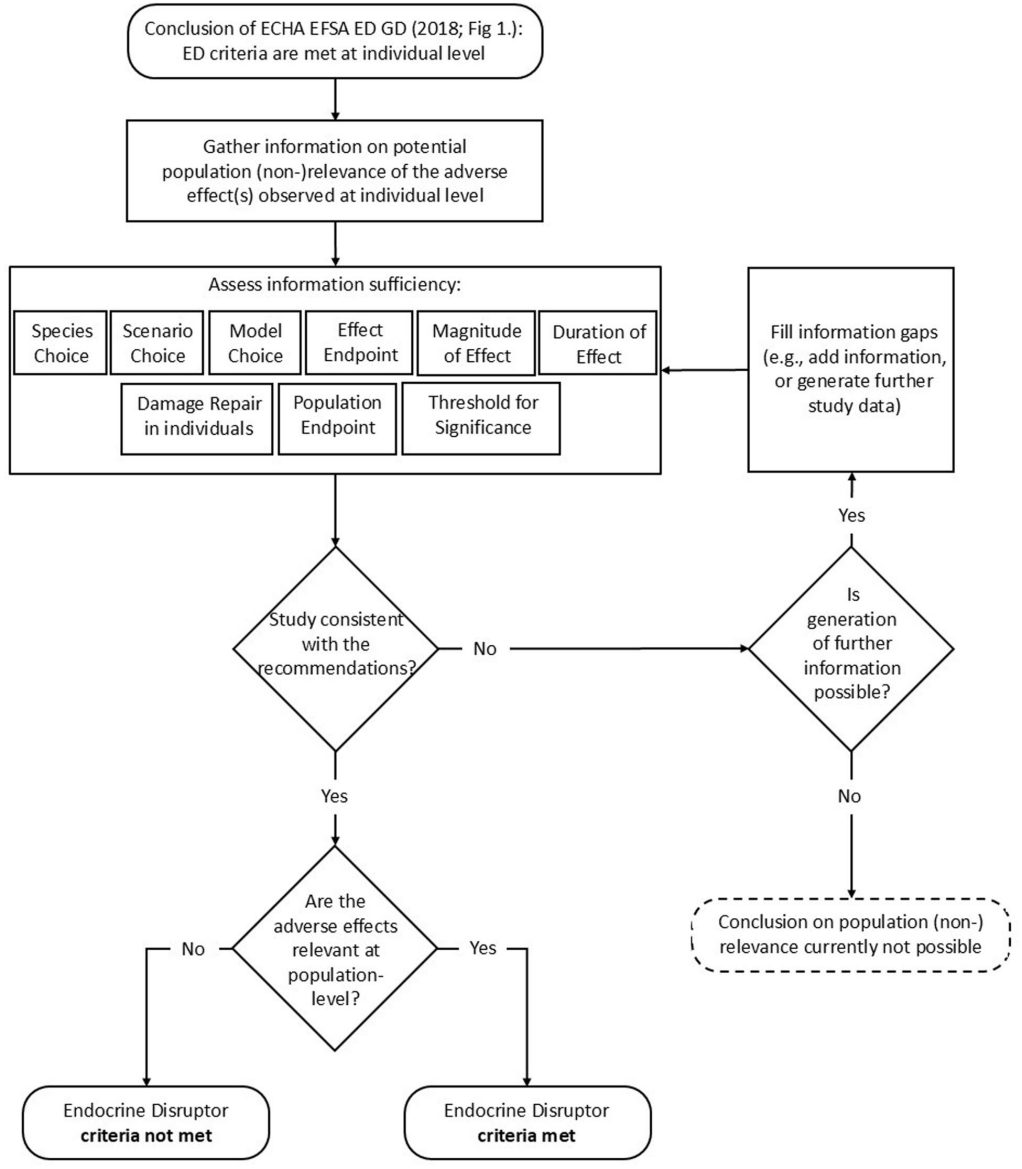
Decision pathway for identifying a substance as an endocrine disruptor according to the assessment strategy proposed by the ECHA & EFSA (2018) guidance and our approach.

Here we have proposed an approach to operationalize the population relevance assessment of EDCs according to the criteria set out in the BP and PPP Regulations (EU) No. 2017/2100 (EC, 2017) and (EU) No. 2018/605 (EC, 2018), respectively (Figure 1). Addressing how models may be parameterized or field studies designed so that they are consistent with the regulation (i.e., specifically, performing a hazard assessment), means that we no longer need to make the (often untrue) simplifying assumption that an adverse effect at the individual level will necessarily translate into an effect at the population level. We believe that a consistent approach across the regulatory science community regarding how this type of assessment should be performed is necessary, hence this proposal. Our review has identified that there are currently no examples in the literature that comply entirely with all aspects of the approach we are recommending. This is not a reflection of the reliability of the reviewed studies but rather a result of modern regulatory standards being imposed on older studies. Future studies could be performed consistently with the proposed approach if that was the aim. The approach has integrated the concepts from a range of different sources and in the short-term, it would be desirable to see examples that fully comply with the approach.

There are still fundamental challenges to using models and field studies that underlie all areas of regulatory assessment. For example, focal species still need to be defined, and appropriate scenarios will need to be parameterized. Without acceptance of baseline models for regulatory use, evaluating the population relevance of adverse effects of EDCs will be challenging. We foresee considerable progress in these areas in the short-term. Application of the proposed approach to monitoring studies requires further consideration. Indeed, due to the nature of monitoring studies to rely on concentrations (and effects) present in the environment, there is no clear way to address the issue of magnitude of effect, and it is difficult to see how monitoring studies could be used routinely in the current hazard-based regulatory context. Equally, the practical and ethical challenges to performing a field study within a hazard context are also considerable, making population modeling the most promising option for assessing the population relevance of EDCs. Although the proposed approach may already be implemented in a regulatory context, it may necessitate a case-by-case evaluation at the current time.

## Supplementary Material

vjae039_Supplementary_Data

## Data Availability

All data are available in this article and the supplementary material.
