# Supplementary material for: Integrating population-level effects into the regulatory assessment of endocrine disrupting substances
Source: Integr Environ Assess Manag. 2025 Jan 6;21(3):639–48. doi: 10.1093/inteam/vjae039 (PMC12047023; doi:10.1093/inteam/vjae039)
Supplement: vjae039_Supplementary_Data [file vjae039_supplementary_data.zip › SI_Pop_Rel_EDC_FINAL_VERSION.docx]

**Supplementary Material Contents**

[Definitions of terminology used throughout the manuscript 1](#_Toc178172626)

[Further background information about assessment of EDCs in other countries / regions 2](#_Toc178172627)

[Definitions of different study types discussed in this manuscript 2](#_Toc178172628)

[Further background information on the study types in a risk (not hazard) assessment context 3](#_Toc178172629)

[Terms used in the literature search 4](#_Toc178172630)

[List of 47 references reviewed in the literature review 6](#_Toc178172631)

[Specific Protection Goals 11](#_Toc178172632)

[Applying the approach: Additional case studies 13](#_Toc178172633)

[Case Study 2: Fish Field Study 13](#_Toc178172634)

[Case study 3: Fish modelling 16](#_Toc178172635)

[Case Study 4: Mammalian (semi) field study 19](#_Toc178172636)

[References 22](#_Toc178172637)

## Definitions of terminology used throughout the manuscript

Throughout this manuscript, we use the term Endocrine Active Chemical (EAC) when discussing chemicals that have an endocrine mechanism, but do not meet the two other criteria (adversity and plausible link). In contrast, an Endocrine Disrupting Chemical (EDC) meets all three criteria (activity, adversity and a plausible link between the two). As the adverse effect could be considered at the individual-level or population-level, whether EAC or EDC is used is context-specific in each case.

## Further background information about assessment of EDCs in other countries / regions

In contrast to the EU (hazard) approach, the programs developed by the United States (US) Environmental Protection Agency (EDSP, Endocrine Disruptor Screening Program) and the Japanese Ministry of the Environment (EXTEND, Extended Tasks on Endocrine Disruption), for example, adopt a tiered risk-based framework (Burden et al., 2022). Furthermore, whilst invertebrates are considered in the testing programme in Japan, in practice the EU and US regulatory assessments focus entirely on vertebrates.

## Definitions of different study types discussed in this manuscript

Monitoring studies combine measurements of chemical concentrations present in the environment with observations of effects on organisms and their populations. Field studies investigate the effects of applied chemical concentrations on individuals and (sub)populations. Population models predict the population responses to an imposed effect occurring at either individual or sub-individual levels. Whilst computational population models come in different forms (Forbes et al., 2009; Accolla et al., 2021) we consider matrix models and Individual-Based Models (IBMs) in this paper. Matrix models structure a population and treat all individuals in a given age/stage cohort the same, thereby reducing model complexity and data needs, whilst IBMs model individuals separately with population dynamics emerging from the interactions of all individuals, making them more complex but potentially more realistic and suitable for our purposes.

## Further background information on the study types in a risk (not hazard) assessment context

Whilst we are dealing with a hazard assessment in the regulation of EDCs, these three methods (population modelling, field studies and monitoring studies) have their regulatory origins in a risk framework for chemical assessment. In this guise, numerous outputs on the use of these methods in chemical risk assessment have been published. For example, the EFSA guidance document “Risk assessment for Birds and Mammals” (EFSA, 2023) includes general considerations on the use of population models and for conducting field studies of PPP for regulatory purposes, and the EFSA “Scientific Opinion on good modelling practice in the context of mechanistic effect models for risk assessment of plant protection products” (EFSA, 2014) provides detailed criteria for developing models for environmental risk assessment evaluations. Raimondo et al. (2021) developed Pop-GUIDE, “Population modelling Guidance, Use, Interpretation, and Development for Ecological Risk Assessment” and a framework for the use of population models in regulatory contexts (Raimondo et al., 2018), whilst Hommen et al. (2016) provide recommendations and case studies for the use of mechanistic effect models for the risk assessment of chemicals. Extensive guidance is available; however, population models are not yet commonly incorporated in the risk assessment of chemicals. A review by Larras et al. (2022) reported that only four population models have been used to support PPP risk assessments between 2011 and 2021 with model reliability of at least one of the four models questioned by the authorities. ECHA & EFSA (2018) allude to the lack of standardised and validated population models, while the representativeness of the specific real-case situation of field and monitoring studies is also often considered dubious (EFSA, 2023). Data, whether generated in the laboratory and used as inputs for IBMs, or generated in field and monitoring studies, must be of high-quality. The availability of these necessary data can depend on the regulatory regime and taxa being studied. For example, the endocrine testing for PPPs and BPs performed in mammals, fish and amphibians is relatively extensive (Day et al., 2018), whereas there are currently no specific endocrine tests validated in the EU for any other vertebrate taxa. Data paucity (such as for low tonnage chemicals under the REACH Regulation (EC) No. 1907/2006; European Parliament and Council, 2006) make reliable model parameterisation fundamentally difficult. Furthermore, for chemicals where an extensive data package is available, complex data interpretation dealing with potentially conflicting data, which can be dependent on the study design and species used (Burden et al., 2022), make model parameterisation challenging. While all three approaches were originally developed with risk-based assessments in mind and have some inherent challenges with regard to these applications, they have been carefully re-considered here for their potential utility in the EU hazard-based framework for EDC assessments.

## Terms used in the literature search

Web of Science (https://www.webofscience.com) was searched with the following string: (“Population-level” OR “Population-relev*” OR “Population-respons*” OR “Population model*” OR “Individual-based model*” OR “Matrix model*” OR “Population stabili*” OR “Population trend*” OR “Field stud*” OR “Field survey*” OR “Field monitoring”) AND (“Endocrine-activ*” OR “Endocrine-disrupt*” OR “Endocrine action” OR “Endocrine modality” OR “Endocrine mechanism” OR “Endocrine mode of action” OR “Endocrine effect*” OR “Endocrine chemical*” OR “androgen*” OR “estrogen*” OR “thyroid” OR “steroid*” OR “EATS”).

Figure S1: The distribution of the results from the literature review into population relevance of endocrine disruptors. The Reviews column includes modelling, field studies and monitoring reviews. The total number (48) exceeds the total of sources reported in the methods section (47), as Burkhardt-Holm et al. (2008) reported results from both monitoring and modelling studies.

## List of 47 references reviewed in the literature review

| **Authors** | **Year** | **Title** | **DOI** |
| --- | --- | --- | --- |
| Hazlerigg, CRE; Mintram, K; Tyler, CR; Weltje L; Thorbek P | 2023 | Harnessing modelling for assessing the population relevance of exposure to endocrine active chemicals | 10.1002/etc.5640 |
| Miller, DH; Villeneuve, DL; Santana-Rodriguez, KJ; Ankley, GT | 2022 | A Multidimensional Matrix Model for Predicting the Effects of Male-Biased Sex Ratios on Fish Populations | 10.1002/etc.5287 |
| Ussery, EJ.; McMaster, ME; Servos, MR; Miller, DH; Munkittrick, KR | 2021 | A 30-Year Study of Impacts, Recovery, and Development of Critical Effect Sizes for Endocrine Disruption in White Sucker (*Catostomus commersonii*) Exposed to Bleached-Kraft Pulp Mill Effluent at Jackfish Bay, Ontario, Canada | 10.3389/fendo.2021.664157 |
| Mintram, KS; Maynard, SK; Brown, AR; Boyd, R; Johnston, ASA; Sibly, RM; Thorbek, P; Tyler, CR | 2020 | Applying a mechanistic model to predict interacting effects of chemical exposure and food availability on fish populations | 10.1016/j.aquatox.2020.105483 |
| Li, Y; Blazer, VS; Iwanowicz, LR; Schall, MK; Smalling, K; Tillitt, DE; Wagner, T | 2020 | Ecological risk assessment of environmental stress and bioactive chemicals to riverine fish populations: An individual-based model of smallmouth bass *Micropterus dolomieu* | 10.1016/j.ecolmodel.2020.109322 |
| Jackson, L; Klerks, P | 2020 | Effects of the synthetic estrogen 17 alpha-ethinylestradiol on *Heterandria formosa* populations: Does matrotrophy circumvent population collapse? | 10.1016/j.aquatox.2020.105659 |
| Crane, M; Hallmark, N; Lagadic, L; Ott, K; Pickford, D; Preuss, T; Thompson, H; Thorbek, P; Weltje, L; Wheeler, JR | 2019 | Assessing the population relevance of endocrine-disrupting effects for nontarget vertebrates exposed to plant protection products | 10.1002/ieam.4113 |
| David, V; Joachim, S; Porcher, JM; Beaudouin, R | 2019 | Modelling BPA effects on three-spined stickleback population dynamics in mesocosms to improve the understanding of population effects | 10.1016/j.scitotenv.2019.07.274 |
| Forbes, VE; Railsback, S; Accolla, C; Birnir, B; Bruins, RJ F; Ducrot, V; Galic, N; Garber, K; Harvey, BC; Jager, HI; Kanarek, A; Pastorok, R; Rebarber, R; Thorbek, P; Salice, CJ | 2019 | Predicting impacts of chemicals from organisms to ecosystem service delivery: A case study of endocrine disruptor effects on trout | 10.1016/j.scitotenv.2018.08.344 |
| Mintram, KS; Brown, AR; Maynard, SK; Thorbek, P; Tyler, CR | 2018 | Capturing ecology in modeling approaches applied to environmental risk assessment of endocrine active chemicals in fish | 10.1080/10408444.2017.1367756 |
| Mintram, KS; Brown, AR; Maynard, SK; Liu, C; Parker, SJ; Tyler, CR; Thorbek, P | 2018 | Assessing population impacts of toxicant-induced disruption of breeding behaviours using an individual-based model for the three-spined stickleback | 10.1016/j.ecolmodel.2018.09.003 |
| Rearick, DC; Ward, J; Venturelli, P; Schoenfuss, H | 2018 | Environmental oestrogens cause predation-induced population decline in a freshwater fish | 10.1098/rsos.181065 |
| Raimondo S, Pollesch N, Kanarek AR & Lehmann DW | 2018 | A framework for linking population model development with ecological risk assessment objectives | [doi.org/10.1002/ieam.2024](https://doi.org/10.1002/ieam.2024) |
| Awkerman, JA.; Raimondo, S | 2018 | Simulated developmental and reproductive impacts on amphibian populations and implications for assessing long-term effects | 10.1016/j.ecoenv.2017.11.047 |
| Marty, MS; Blankinship, A; Chambers, J; Constantine, L; Kloas, W; Kumar, A; Lagadic, L; Meador, J; Pickford, D; Schwarz, T; Verslycke, T | 2017 | Population-Relevant Endpoints in the Evaluation of Endocrine-Active Substances (EAS) for Ecotoxicological Hazard and Risk Assessment | 10.1002/ieam.1887 |
| Conolly RB, Ankley GT, Cheng W et al. | 2017 | Quantitative Adverse Outcome Pathways and their application to predictive toxicology | 10.1021/acs.est.6b06230 |
| Schwindt, AR; Winkelman, DL | 2016 | Estimating the effects of 17 alpha-ethinylestradiol on stochastic population growth rate of fathead minnows: a population synthesis of empirically derived vital rates | 10.1007/s10646-016-1688-9 |
| Pavlova, V; Grimm, V; Dietz, R; Sonne, C; Vorkamp, K; Riget, FF; Letcher, RJ; Gustavson, K; Desforges, JP; Nabe-Nielsen, J | 2016 | Modeling Population-Level Consequences of Polychlorinated Biphenyl Exposure in East Greenland Polar Bears | 10.1007/s00244-015-0203-2 |
| Topping, CJ; Dalby, L; Skov, F | 2016 | Landscape structure and management alter the outcome of a pesticide ERA: Evaluating impacts of endocrine disruption using the ALMaSS European Brown Hare model | 10.1016/j.scitotenv.2015.10.042 |
| Miller, DH; Tietge, JE; McMaster, ME; Munkittrick, KR; Xia, X; Griesmer, DA; Ankley, GT | 2015 | Linking mechanistic toxicology to population models in forecasting recovery from chemical stress: A case study from Jackfish Bay, Ontario, Canada | 10.1002/etc.2972 |
| Hazlerigg, CRE; Tyler, CR; Lorenzen, K; Wheeler, JR; Thorbek, P | 2014 | Population relevance of toxicant mediated changes in sex ratio in fish: An assessment using an individual-based zebrafish (*Danio rerio*) model | 10.1016/j.ecolmodel.2013.12.016 |
| McNair, AS; Nakagawa, S; Grimm, V | 2014 | The Evolutionary Consequences of Disrupted Male Mating Signals: An Agent-Based Modelling Exploration of Endocrine Disrupting Chemicals in the Guppy | 10.1371/journal.pone.0103100 |
| Brown, AR; Gunnarsson, L; Kristiansson, E; Tyler, CR | 2014 | Assessing variation in the potential susceptibility of fish to pharmaceuticals, considering evolutionary differences in their physiology and ecology | 10.1098/rstb.2013.0576 |
| Miller, DH; Tietge, JE; McMaster, ME; Munkittrick, KR; Xia, X; Ankley, GT | 2013 | Assessment of status of white sucker (*Catostomus commersoni*) populations exposed to bleached kraft pulp mill effluent | 10.1002/etc.2218 |
| Brander, SM; Connon, RE; He, G; Hobbs, JA; Smalling, KL; Teh, SJ; White, JW; Werner, I; Denison, MS; Cherr, GN | 2013 | From 'Omics to Otoliths: Responses of an Estuarine Fish to Endocrine Disrupting Compounds across Biological Scales | 10.1371/journal.pone.0074251 |
| Dalkvist, T; Sibly, RM; Topping, CJ. | 2013 | Landscape structure mediates the effects of a stressor on field vole populations | 10.1007/s10980-013-9932-7 |
| Chiang, G; McMaster, ME; Urrutia, R; Fernanda SM; Francisco GJ; Tucca, F; Barra, R; Munkittrick, KR | 2011 | Health status of native fish (*percilia gillissi* and *trichomycterus areolatus*) downstream of the discharge of effluent from a tertiary-treated elemental chlorine-free pulp mill in chile | 10.1002/etc.573 |
| Raimondo, S; Hemmer, BL; Goodman, LR; Cripe, GM | 2009 | Multigenerational exposure of the estuarine sheepshead minnow (*cyprinodon variegatus*) to 17 beta-estradiol. ii. population-level effects through two life cycles | 10.1897/08-540.1 |
| Dalkvist, T; Topping, CJ; Forbes, VE | 2009 | Population-level impacts of pesticide-induced chronic effects on individuals depend more on ecology than toxicology | 10.1016/j.ecoenv.2008.10.002 |
| Palace, V. P.; Evans, R. E.; Wautier, K. G.; Mills, K. H.; Blanchfield, P. J.; Park, B. J.; Baron, C. L.; Kidd, K. A. | 2009 | Interspecies differences in biochemical, histopathological, and population responses in four wild fish species exposed to ethynylestradiol added to a whole lake | 10.1139/F09-125 |
| Ankley, GT; Miller, DH; Jensen, KM; Villeneuve, DL; Martinovic, D | 2008 | Relationship of plasma sex steroid concentrations in female fathead minnows to reproductive success and population status | 10.1016/j.aquatox.2008.03.005 |
| Burkhardt-Holm, P; Segner, H; Burki, R; Peter, A; Schubert, S; Suter, MJF; Borsuk, ME | 2008 | Estrogenic endocrine disruption in Switzerland: Assessment of fish exposure and effects | 10.2533/chimia.2008.376 |
| Green, RE; Taggart, MA; Senacha, KR; Raghavan, B; Pain, DJ; Jhala, Y; Cuthbert, R | 2007 | Rate of Decline of the Oriental White-Backed Vulture Population in India Estimated from a Survey of Diclofenac Residues in Carcasses of Ungulates | 10.1371/journal.pone.0000686 |
| Kidd, KA; Blanchfield, PJ; Mills, KH; Palace, VP; Evans, RE; Lazorchak, JM; Flick, RW | 2007 | Collapse of a fish population after exposure to a synthetic estrogen | 10.1073/pnas.0609568104 |
| Gutjahr-Gobell, RE; Zaroogian, GE; Horowitz, DJB; Gleason, TR; Mills, LJ | 2006 | Individual effects of estrogens on a marine fish, Cunner (*Tautogolabrus adspersus*), extrapolated to the population level | 10.1016/j.ecoenv.2005.05.017 |
| Gurney, William S. C. | 2006 | Modeling the demographic effects of endocrine disruptors | 10.1289/ehp.8064 |
| Palace, VP; Wautier, KG; Evans, RE; Blanchfield, PJ; Mills, KH; Chalanchuk, SM; Godard, D; McMaster, ME; Tetreault, GR; Peters, LE; Vandenbyllaardt, L; Kidd, KA | 2006 | Biochemical and histopathological effects in pearl dace (*Margariscus margarita*) chronically exposed to a synthetic estrogen in a whole lake experiment | 10.1897/04-557R1.1 |
| Werner, J; Palace, VP; Wautier, KG; Mills, KH; Chalanchuk, SM; Kidd, KA | 2006 | Reproductive fitness of lake trout (*Salvelinus namaycush*) exposed to environmentally relevant concentrations of the potent estrogen ethynylestradiol (EE2) in a whole lake exposure experiment | 10.3989/scimar.2006.70s259 |
| West, DW; Ling, N; Hicks, BJ; Tremblay, LA; Kim, ND; van den Heuvel, MR | 2006 | Cumulative impacts assessment along a large river, using brown bullhead catfish (*Ameiurus nebulosus*) populations | 10.1897/05-315R.1 |
| Segner, H | 2005 | Developmental, reproductive, and demographic alterations in aquatic wildlife: Establishing causality between exposure to endocrine-active compounds (EACs) and effects | 10.1002/aheh.200400550 |
| Hanson, N; Aberg, P; Sundelof, A | 2005 | Population-level effects of male-biased broods in eelpout (*Zoarces viviparus*) | 10.1897/04-185R.1 |
| Brown, AR; Riddle, AM; Winfield, IJ; Fletcher, JM; James, JB | 2005 | Predicting the effects of endocrine disrupting chemicals on healthy and disease impacted populations of perch (*perca fluviatilis*) | 10.1016/j.ecolmodel.2005.03.009 |
| Miller, DH; Ankley, GT | 2004 | Modeling impacts on populations: fathead minnow (*Pimephales promelas*) exposure to the endocrine disruptor 17 beta-trenbolone as a case study | 10.1016/j.ecoenv.2004.05.005 |
| Brown, AR; Riddle, AM; Cunningham, NL; Kedwards, TJ; Shillabeer, N; Hutchinson, TH | 2003 | Predicting the effects of endocrine disrupting chemicals on fish populations | 10.1080/713609966 |
| Gleason, TR; Nacci, DE | 2001 | Risks of endocrine-disrupting compounds to wildlife: Extrapolating from effects on individuals to population response | 10.1080/20018091094835 |
| Bowerman, WW; Best, DA; Grubb, TG; Sikarskie, JG; Giesy, JP | 2000 | Assessment of environmental endocrine disruptors in bald eagles of the Great Lakes | 10.1016/S0045-6535(00)00014-X |
| Caslin, TM; Wolff, JO | 1999 | Individual and demographic responses of the gray-tailed vole to vinclozolin | 10.1002/etc.5620180727 |

## Specific Protection Goals

The proposed SPGs for the population relevance of EDCs are presented in Table S1. The selections proposed are consistent with the ethos of a hazard assessment as required in EU Regulations (EC) No. 1107/2009 (European Parliament and Council, 2009) and (EU) No. 528/2012 (European Parliament and Council, 2012). Generally, when the focus is on long-term population dynamics a longer temporal scale is more appropriate (EFSA, 2016), hence the months-seasons (or species-specific “generations”) temporal scale is recommended here, as relevant to the organisms of the assessment (terrestrial and aquatic vertebrates). This means that an exposed population may deviate in abundance / biomass from an unexposed population for a short duration (e.g., days) and this would not be considered an adverse population effect (note this is not the same as population recovery, because the adverse effect has not yet been observed). This is also consistent with how population responses to stressors are commonly evaluated, with census on a given day of the year being one such metric (e.g., Mintram et al., 2018a). Meanwhile, the recommended spatial scale should be consistent with the entity to be protected, so rather than a unit of area, a spatial scale associated with a given population is proposed. This is dependent upon the species under investigation, though defining where one population ends and another begins can be challenging, especially for mobile species (EFSA, 2016).

Table S1: Proposed Specific Protection Goal for population relevance of EDCs

| Dimension | Selection | Reasoning |
| --- | --- | --- |
| Ecological entity | Population | Identified in Regulation (EU) No. 2018/605 (EC, 2018) |
| Attribute | Abundance, Biomass, Size distribution | Selection proposed by Crane et al. (2019), see reference for more information |
| Magnitude | Normal Operating Range (of the population) | Identified in Regulation (EU) No. 2018/605 (EC, 2018): this is a hazard assessment so recovery is not acceptable making any effect unacceptable |
| Temporal scale | Months, Seasons, Generations | EFSA (2016) states that “seasons to rotations are relevant when the temporal scale of effects has its focus on long-term population dynamics, including risk of local extinction”. Alternatively, to observe a population response the time must cover multiple generations (this would then be shorter or longer depending on the selected species). |
| Spatial scale | Population range | Instead of proposing a specific area size, we propose to define the spatial scale on the range of the investigated population. This is consistent with the other dimensions of this SPG, though requires the definition of a population as well as requiring different spatial scales depending on the selected species. |

EDC – endocrine disrupting chemical, SPG – specific protection goal

## Applying the approach: Additional case studies

### Case Study 2: Fish Field Study

*Study description:* A 7-year whole-lake study exploring the effects of EE2 on 4 different fish species (fathead minnow, *Pimephales promelas*, pearl dace, *Margariscus margarita*, lake trout, *Salvelinus namaycush* and white sucker, *Catostomus commersonii*) was performed at the Experimental Lakes Area (Ontario, Canada) from 1999-2005 (Palace et al., 2006; Werner et al., 2006; Kidd et al., 2007; Palace et al., 2009). An initial 2 years of study to confirm baseline fish population levels in the target lake and two reference lakes was performed, followed by a 3-year period with addition of EE2 and then a further 2-year recovery period without EE2 additions. EE2 concentrations were maintained around levels known to occur in monitored waterways associated with wastewater treatment plants (approx. 5-6 ng/L). EE2 was added thrice weekly during the non-frozen period (20-22 weeks/year), with dosage dependent on analytical sampling from the preceding week. A range of fish traps were installed in the autumn and spring to collect fish for assessment of individual-level parameters (e.g., length, weight, histology etc.) as well as to determine population responses (i.e., abundance via Catch Per Unit Effort and size distributions). These metrics indicated the population of fathead minnow declined in the second year of EE2 application. The population of pearl dace and lake trout declined later in the third year of EE2 application, whilst the population of white sucker did not decline over the entire study period.

*Implementation of the 7 points of the proposed approach:* The four fish species studied were all native to the lake site under investigation. As such, they may be considered potentially exposed to EE2 in that environmental site. Given this was a field study, some form of exposure was required in order to assess the effects of an EDC. The authors implemented concentrations of EE2 in the lake that were consistent with those concentrations that had been observed in other waterways with known high contamination status (as EE2 equivalents). The EE2 was applied thrice weekly (during ice-free seasons) for three years. Any damage repair would be a result of individual-level detoxification processes and any variation in exposure between micro-habitats. Population abundances and size distributions were monitored based on catches, with trends analysed to show population crashes, with the threshold for a population response the result of statistical tests.

*Conclusions for regulatory use:* Whilst this case study implements some of the recommendations within this manuscript, further work would be required to use this in a regulatory context. Firstly, there were other species present in the lake during the experiment (in addition to the four studied). This study does illustrate that multiple species may be tested in a given field experiment, which can be particularly useful when different feeding guilds are present and it is not clear which species may be the most vulnerable *a priori*. For example, other small-bodied fish were present in the lake, however, pearl dace was chosen because it is a primary forage fish species due to its short reproductive cycle (2-3 years), and the endpoints being investigated could all be measured with a reasonable measure of precision. Meanwhile the large-bodied species (lake trout and white sucker) represented species with different life histories and their longer generation time was one reason suggested for the limited population-level effect observed in the study (as the exposure only lasted for three years). Finally, the fathead minnow is a temperate, wide-spread, fractional spawning species that is an established laboratory test species due to its ease of handling, tolerance to a wide range of basic water quality characteristics and relatively short generation time (Ankley & Villeneuve, 2006). As such, it is potentially a relevant model species, however, it may be more appropriate for North America, as it is not present in EU waters. European equivalents with similar life histories could likely be found to perform a study relevant to European habitat, however the need to find a suitable site for such a study in Europe may also present a challenge, so further work on defining the scenario would also be required. Whilst theoretically possible and of considerable value in understanding the population relevance of a potential EDC, regular use of this type of large-scale study will face significant regulatory hurdles in being granted authorisation to proceed given current strict animal welfare standards and unified ambition to reduce animal testing generally. A small-scale, semi-field case study may be more likely to be used on a regular basis.

Secondly, EE2 was applied at concentrations observed in monitoring studies. This would not be appropriate for a field study designed to assess the population relevance of EDCs under the PPP or BP Regulations ((EC) No. 1107/2009, European Parliament and Council (2009); (EU) No. 528/2012, European Parliament and Council (2012)) as these require prospective assessment and as such, monitoring levels may not be consistent with the proposed uses (and hence, exposures) of a product. Furthermore, the exposure concentration of EE2 was not associated with any study-derived MTC, however a review by Caldwell et al. (2012) reported observable generalised toxicity in studies with Chinese rare minnow (reduced survival in F1 generation) at 0.2 ng/L and zebrafish (reduced length and weight) at 2 ng/L. This indicates that the 5-6 ng/L used in this study might have exceeded the MTC and a lower exposure should be used when considering ED-mediated effects only.

Finally, the EE2 exposure duration of 3 years may or may not be considered appropriate for regulatory purposes and should be considered on a case-by-case basis (e.g. we propose a 1-year duration in modelling studies unless underlying assumptions cannot be justified). These inconsistencies with the approach proposed in this manuscript could be addressed in future (semi) field studies, whilst the rest of the field study was consistent with the approach proposed – namely, suitable selection of individual-level endpoints and appropriate population level attributes to assess. Whilst the costs of performing such a study are considerable, these could be reduced as there would be no regulatory need to continue the study for 2 years after exposure ceased as population recovery is not an option.

### Case study 3: Fish modelling

*Study description:* Conolly et al. (2017) used a density-dependent matrix model to link chemical effects on egg development and spawning of fathead minnow (*Pimephales promelas*) with a 10-year trajectory in population size. Effects of fadrozole, an aromatase inhibitor, on egg development and spawning were taken from a 21-day Fish Short Term Reproduction laboratory test (Ankley et al., 2002). The effects observed at each tested concentration were then inputted into the matrix model, to investigate the population response following exposure to different concentrations of fadrozole. The study showed a reduction in fecundity (eggs/female/day) of 15% was associated with a reduction in population size of 10%.

*Implementation of the 7 points of the proposed approach:* The study uses the fathead minnow, a well-established laboratory test species. The study considers a matrix model that had been developed previously for the fathead minnow. The study considers effects on fecundity with the magnitude of effect imposed in the model based on the dose-response relationship in the 21-day laboratory test of Ankley et al. (2002). The effect was imposed continuously for a period of 10 years, with no damage repair of effected individuals. The population size was reported as a proportion of the carrying capacity. The authors consider a 20% reduction in fathead minnow population as a threshold for effects.

*Conclusions for regulatory use:* Whilst this case study implements some of the recommendations within this manuscript, further work would be required to use this in a regulatory context.

Considerable discussion on the suitability of fish species (including the appropriateness of the fathead minnow) is provided in the first case study and not repeated here. Regarding model structure there are numerous population model types of relevance for risk assessment available for vertebrates as discussed earlier. Matrix models are one of the most common models used to simulate population-level effects of chemicals, with the approach having extensive benefits (e.g. relatively few data for parameterisation, intuitive metric on time to extinction that can be linked to a specific protection goal). However, they typically do not consider environmental variability and thus are less realistic (Mintram et al., 2018b). Other modelling approaches can more easily be made to include structural and spatial complexity and multiple parameters, allowing incorporation of life-history strategies, interactions between individuals and ecological processes (e.g. individual-based models (IBMs)). This has led to previous publications concluding that IBMs are deemed the most useful population models for the environmental risk assessment of chemicals (Accolla et al., 2021; Crane et al., 2019; Mintram et al., 2018b). For fish, the three-spined stickleback (*Gasterosteus aculeatus*) IBM (Mintram et al., 2018a; 2020), the brown trout (*Salmo trutta*) Energy Budget-IBM called inSTREAM (Forbes et al., 2019; Railsback et al., 2009) and the zebrafish (*Danio rerio*) IBM (Hazlerigg et al., 2014) have been used to assess population relevance of EDCs and may be considered acceptable for use in chemical hazard assessments based on EFSA’s Good Modelling Practice guidelines (EFSA, 2014). Nevertheless, there are trade-offs with all models and as such, the choice of model type should be informed by the question to be answered. In this instance, either model type might be used, but an IBM may prove invaluable to further understand the biological and ecological processes involved and support the regulatory acceptance of the results.

Next, this example assumes that fadrozole has already been confirmed as an EDC at the individual level and that the only endpoint adversely effected by exposure to fadrozole is fecundity. Fecundity is one of the individual-level apical endpoints relevant at the population-level recommended by Marty et al. (2017). Though note that Marty et al. (2017) also identified growth (body weight, length), hatching success, sex ratio and offspring survival as potentially relevant at the population-level and this simplified case study did not consider these in the model. As such, consideration of fecundity alone is a simplification to illustrate the method. For regulatory use, a chronic higher tier study would be more appropriate (thorough) to determine the long-term effects of a potential EDC and integrate them across all life-stages (for example, fadrozole is also known to affect sex ratio; Andersen et al., 2004).

Finally, effects were imposed according to a dose-response relationship. However, to be consistent with a hazard-based assessment of EDCs, we recommend the use of the magnitude of effect measured at the MTC. However, in Ankley et al. (2002), a significant effect on fecundity was observed at all tested concentrations (i.e. the lowest tested concentration of 2 µg/L (nominal, 1.4 µg/L measured concentration) was associated with a fecundity decline of about 43% compared to the control) and no MTC was determined. For any regulatory use, a study with a defined MTC should be used. For example purposes only, in a regulatory hazard-based assessment of fadrozole using the magnitude of effect on individuals of 43%, the outcome of Conolly et al. (2017) would result in the chemical failing the assessment as dramatic population effects were observed.

### Case Study 4: Mammalian (semi) field study

*Study description:* Caslin & Wolff (1999) performed two enclosure studies exploring the effects of exposure to the vinclozolin-based fungicide product Curalan on gray-tailed voles (*Microtus canicaudus*). The first was performed in the wet season, whilst the second was performed in the dry season. In the first study, five male and between seven to ten female voles were introduced into eight 0.5 acre enclosures planted with a mixture of pasture grasses to a height of 50-60cm. Three weeks after vole introductions (timed to coincide with females being pregnant), all males were removed from the enclosures and the four treatment enclosures received a single application of 12.9 L of Curalan (the rate recommended on the product label), whilst the four control enclosures received an equivalent application of water. This was applied using a tractor and trailer tank with 11m spray booms. Within 24 hours of application, the enclosures received 5.4 mm of rainfall. Every two weeks, 9 traps per enclosure were baited and information on captured individuals was recorded (weight, sex, location, reproductive condition). Population size was estimated by the minimum number of voles known to be alive, whilst recruitment was calculated as the number of new recruits caught per female in the initial population. From each enclosure five male recruits (who had been exposed) were assessed for indications of toxicity. Some metrics indicated a treatment-related effect on males (e.g., mean testes size was reduced in the treated groups, plasma testosterone was significantly lower than in the control group), however, living sperm was still present in all males sampled. The number of recruits per adult female was slightly (but not significantly) lower in the treated groups (3.6 compared with 3.8) as were the mean peak population sizes (28.8 compared to 29.8 individuals) indicating no meaningful population level effect from exposure to Curalan. The second study was performed in a similar way, but with shorter grass (20-30cm) and without the additional rainfall after application. The results of the second study were consistent with those from the first study.

*Implementation of the 7 points of the proposed approach:* The study used the gray-tailed vole, a commonly used terrestrial mammal in studies conducted in North America. The study site consisted of small, grass-covered enclosures where gray-tailed voles would commonly be found. The study specifically focussed on pregnant females with the effect endpoint, magnitude of effect and duration of effect the result of exposure to Curalan sprayed on the enclosure at the representative product rate. Exposure was not maintained throughout the study, so damage repair was dependent upon natural processes of chemical decline in the environment, the behaviour of individuals and their internal detoxification processes. Population abundance was reported, though no specific threshold for significant effects nor statistical testing was performed.

*Conclusions for regulatory use:* Whilst this case study implements some of the recommendations within this manuscript, further work would be required to use this in a regulatory context.

Considerable discussion on the suitability of different mammalian species is provided in the Dalkvist et al. (2009) case study in the main paper and not repeated here. Whilst the vole species is different from that in the second case study, similar conclusions regarding its presence in agricultural landscapes and potential vulnerability can be made. The authors had also used gray-tailed voles in previous studies making the husbandry considerations more manageable. The enclosures were relatively small with an initial population of only 12-15 voles and recruited population of only 29.8 voles. This is unlikely to be representative of a vole population in the wild, meaning other population regulation mechanisms relating to competition for resources, anti-predation behaviours etc. were absent from this study.

Next, the exposure concentration was not associated with any study-derived MTD and furthermore, the decay rates of vinclozolin are known to be highly variable among plant types and in response to sunlight, temperature and rainfall making it impossible to accurately state the exposure (and hence, effect) to any individual vole in the enclosure. Whilst no evidence of generalised toxicity was reported in the analyses, suggesting the rate may not exceed the MTD, it is not possible to determine whether the rate was too low (i.e., below the MTD) and therefore under-estimating potential endocrine-mediated effects on the voles. As such, this approach would not necessarily be considered appropriate for a field study designed to assess the population relevance of EDCs under the PPPs or BPs Regulations ((EC) No. 1107/2009, European Parliament and Council (2009); (EU) No. 528/2012, European Parliament and Council (2012)). The exposure duration of up to 2 months (depending on the rate of residue decline after application) may or may not be considered appropriate for regulatory purposes and should be considered on a case-by-case basis. In this case, the study duration may be suitable to investigate the effects of vinclozolin exposure during pregnancy, however, this assumes no other ED-mediated effects are associated with the substance (which is not the case) and that no carry-over effects exist (which is unlikely).

A suitable population endpoint (abundance) was chosen for assessment, though the differences were not analysed statistically because of the removal of some animals for necropsy so no threshold for effect was set. A note of caution when evaluating (semi-) field studies that due to inherently high variance statistical approaches require the study to have suitable power to detect differences.

## References

Accolla, C., Vaugeois, M., Grimm, V., Moore, A. P., Rueda‐Cediel, P., Schmolke, A., & Forbes, V. E. (2021). A review of key features and their implementation in unstructured, structured, and agent‐based population models for ecological risk assessment. *Integrated environmental assessment and management*, 17(3), 521-540. https://doi.org/10.1002/ieam.4362

Andersen, L., Kinnberg, K., Holbech, H., Korsgaard, B., Bjerregaard, P. (2004). Evaluation of a 40 day assay for testing endocrine disrupters: effects of an anti-estrogen and an aromatase inhibitor on sex ratio and vitellogenin concentrations in juvenile zebrafish (*Danio rerio*). *Fish Physiology and Biochemistry*, 30, 257–266.

Ankley, G. T., Kahl, M. D., Jensen, K. M., Hornung, M. W., Korte, J. J., Makynen, E. A., Leino, R. L. (2002). Evaluation of the aromatase inhibitor fadrozole in a short-term reproduction assay with the fathead minnow (*Pimephales promelas*). *Toxicological Sciences*, 67, 121−130. https://doi-org/10.1093/toxsci/67.1.121.

Ankley, G. T. & Villeneuve, D. L. (2006). The fathead minnow in aquatic toxicology: Past, present and future. *Aquatic Toxicology*, 78, 91-102. https://doi-org/10.1016/j.aquatox.2006.01.018

Burden, N., Embry, M. R., Hutchinson, T. H., Lynn, S. G., Maynard, S. K., Mitchell, C. A., Pellizzato, F., Sewell, F., Thorpe, K.L., Weltje, L. and Wheeler, J.R. (2022). Investigating endocrine‐disrupting properties of chemicals in fish and amphibians: Opportunities to apply the 3Rs. *Integrated Environmental Assessment and Management*, 18(2), 442-458. https://doi.org/10.1002/ieam.4497

Caldwell, D. J., Mastrocco, F., Anderson, P. D., Lange, R. & Sumpter, J. P. (2012). Predicted-no-effect concentrations for the steroid estrogens estrone, 17β-estradiol, estriol and 17α-ethinylestradiol. *Environmental Toxicology & Chemistry*, 31(6), 1396-1406.  https://doi.org/10.1002/etc.1825

Caslin, T. M. & Wolff, J. O. (1999). Individual and demographic responses of the gray-tailed vole to vinclozolin. *Environmental Toxicology and Chemistry,* 18(7), 1529-1533. https://doi.org/10.1002/etc.5620180727

Conolly, R.B., Ankley, G.T., Cheng, W., Mayo, M.L., Miller, D.H., Perkins, E.J., Villeneuve, D.L. & Watanabe, K.H., (2017). Quantitative adverse outcome pathways and their application to predictive toxicology. Environmental Science & Technology, 51(8), 4661-4672. https://doi.org/10.1021/acs.est.6b06230

Crane, M., Hallmark, N., Lagadic, L., Ott, K., Pickford, D., Preuss, T., Thompson, H., Thorbek, P., Weltje, L. & Wheeler, J. R. (2019). Assessing the population relevance of endocrine-disrupting effects for nontarget vertebrates exposed to plant protection products. *Integrated Environmental Assessment & Management*, 15(2), 278-291. https://doi.org/10.1002/ieam.4113

Dalkvist, T., Topping, C. J. & Forbes, V. E. (2009). Population-level impacts of pesticide-induced chronic effects on individuals depend more on ecology than toxicology. Ecotoxicology and Environmental Safety, 72, 1663-1672. https://doi.org/10.1016/j.ecoenv.2008.10.002

Day, P., Green, R.M., Gross, M., Weltje, L. & Wheeler, J.R. (2018) Endocrine disruption: current approaches for regulatory testing and assessment of plant protection products are fit for purpose. *Toxicology Letters*, 296, 10-22. https://doi.org/10.1016/j.toxlet.2018.07.011

EC (2018). COMMISSION REGULATION (EU) 2018/605 of 19 April 2018 amending Annex II to Regulation (EC) No 1107/2009 by setting out scientific criteria for the determination of endocrine disrupting properties. *Official Journal of the European Union* L 101/33.

ECHA (European Chemicals Agency) and EFSA (European Food Safety Authority) with the technical support of the Joint Research Centre (JRC), Andersson, N., Arena, M., Auteri, D., Barmaz, S., Grignard, E., Kienzler, A., Lepper, P., Lostia, A. M., Munn, S., Parra Morte, J. M., Pellizzato, F., Tarazona, J., Terron, A. & Van der Linder, S. (2018). Guidance for the identification of endocrine disruptors in the context of Regulations (EU) No 528/2012 and (EC) No 1107/2009. *EFSA Journal*, 16(6), 5311. https://doi.org/10.2903/j.efsa.2018.5311

EFSA (2014) Scientific Opinion on good modelling practice in the context of mechanistic effect models for risk assessment of plant protection products. *EFSA Journal*, 12(3), 3589. https://doi.org/10.2903/j.efsa.2014.3589

EFSA (2016). Guidance to develop specific protection goals options for environmental risk assessment at EFSA, in relation to biodiversity and ecosystem services. *EFSA Journal*, 14(6), e04499. https://doi.org/10.2903/j.efsa.2016.4499

EFSA (2023) Risk assessment for Birds and Mammals. *EFSA Journal* 21(2): e07790.   https://doi.org/10.2903/j.efsa.2023.7790

European Parliament and Council (2006). Regulation (EC) No 1907/2006 of the European Parliament and of the Council of 18 December 2006 concerning the Registration, Evaluation, Authorisation and Restriction of Chemicals (REACH), establishing a European Chemicals Agency, amending Directive 1999/45/EC and repealing Council Regulation (EEC) No 793/93 and Commission Regulation (EC) No 1488/94 as well as Council Directive 76/769/EEC and Commission Directives 91/155/EEC, 93/67/EEC, 93/105/EC and 2000/21/EC. *Official Journal of the European Union* L396.

European Parliament and Council (2009). Regulation (EC) No 1107/2009 of the European Parliament and of the Council of 21 October 2009 concerning the placing of plant protection products on the market and repealing Council Directives 79/117/EEC and 91/414/EEC. *Official Journal of the European Union* L101/33

European Parliament and Council (2012). Regulation (EU) No 528/2012 of the European Parliament and of the Council of 22 May 2012 concerning the making available on the market and use of biocidal products Text with EEA relevance. *Official Journal of the European Union* L167.

Forbes, V. E., Calow, P. & Sibly, R. M. (2009). The extrapolation problem and how population modelling can help. *Environmental Toxicology & Chemistry,* 27(10), 1987-1994. https://doi.org/10.1897/08-029.1

Forbes, V. E., Railsback, S., Accolla, C., Birnir, B., Bruins, R. J. F., Ducrot, V., Galic, N. *et al.* (2019). Predicting impacts of chemicals from organisms to ecosystem service delivery: A case study of endocrine disruptor effects on trout. *Science of the Total Environment,* 649, 949-959. https://doi.org/10.1016/j.scitotenv.2018.08.344

Hazlerigg, C. R. E., Tyler, C. R., Lorenzen, K., Wheeler, J. R. & Thorbek, P. (2014). Population relevance of toxicant mediated changes in sex ratio in fish: An assessment using an individual-based zebrafish (*Danio rerio*) model. *Ecological Modelling*, 280, 76-88. https://doi.org/10.1016/j.ecolmodel.2013.12.016

Hommen, U., Forbes, V., Grimm, V., Preuss, T. G., Thorbek, P., & Ducrot, V. (2016). How to use mechanistic effect models in environmental risk assessment of pesticides: case studies and recommendations from the SETAC workshop MODELINK. *Integrated environmental assessment and management*, 12(1), 21-31. https://doi.org/10.1002/ieam.1704

Kidd, K. A., Blanchfield, P. J., Mills, K. H., Palace, V. P., Evans, R. E., Lazorchak, J. M. & Flick, R. W. (2007). Collapse of a fish population after exposure to a synthetic estrogen. Proceedings of the National Academy of Sciences, 104(21), 8897-8901. https://doi.org/10.1073/pnas.0609568104

Larras, F., Charles, S., Chaumot, A., Pelosi, C., Le Gall, M., Mamy, L. & Beaudouin, R. (2022). A critical review of effect modelling for ecological risk assessment of plant protection products. *Environmental Science and Pollution Research,* 29, 43448-43500. https://doi.org/10.1007/s11356-022-19111-3

Marty, M. S., Blankinship, A., Chambers, J., Constantine, L., Kloas, W., Kumar, A., Lagadic, L., Meador, J., Pickford, D., Schwarz, T. & Verslycke, T. (2017). Population-relevant endpoints in the evaluation of endocrine-active substances (EAS) for ecotoxicological hazard and risk assessment. *Integrated Environmental Assessment and Management*, 13(2), 317-330. https://doi.org/10.1002/ieam.1887

Mintram, K. S., Brown, A. R., Maynard, S. K., Liu, C., Parker, S. J., Tyler, C. R. & Thorbek, P. (2018a). Assessing population impacts of toxicant-induced disruption of breeding behaviours using an individual-based model for the three-spined stickleback. *Ecological Modelling*, 387, 107-117. https://doi.org/10.1016/j.ecolmodel.2018.09.003

Mintram, K. S., Brown, A. R., Maynard, S. K., Thorbek, P., & Tyler, C. R. (2018b). Capturing ecology in modeling approaches applied to environmental risk assessment of endocrine active chemicals in fish. *Critical Reviews in Toxicology*, 48(2), 109-120. https://doi.org/10.1080/10408444.2017.1367756

Mintram, K. S., Maynard, S. K., Brown, A. R., Johnston, A. S. A., Sibley, R. M., Thorbek, P. & Tyler, C. R. (2020). Applying a mechanistic model to predict interacting effects of chemical exposure and food availability on fish populations. *Aquatic Toxicology*, 224, 105483. https://doi.org/10.1016/j.aquatox.2020.105483

Palace, V. P., Wautier, K. G., Evans, R. E., Blanchfield, P. J., Mills, K. H., Chalanchuk, S. M., Godard, D., McMaster, M. E., Tetreault, G. R., Peters, L. E., Vandenbyllaardt, L. & Kidd, K. A. (2006). Biochemical and histopathological effects in pearl dace (*Margariscus margarita*) chronically exposed to a synthetic estrogen in a whole lake experiment. *Environmental Toxicology and Chemistry,* 25(4), 1114-1125. https://doi.org/10.1897/04-557R1.1

Palace, V. P., Evans, R. E., Wautier, K. G., Mills, K. H., Blanchfield, P. J., Park, B. J., Baron, C. L. & Kidd, K. A. (2009). Interspecies differences in biochemical, histopathological and population response in four wild fish species exposed to ethynylestradiol added to a whole lake. *Canadian Journal of Fisheries & Aquatic Science*, 66, 1920-1935. https://doi.org/10.1139/F09-125

Railsback, S. F., Harvey, B. C., Jackson, S. K., & Lamberson, R. H. (2009). InSTREAM: the individual-based stream trout research and environmental assessment model (p. 254). Albany: US Department of Agriculture, Forest Service, Pacific Southwest Research Station.

Raimondo, S., Etterson, M., Pollesch, N., Garber, K., Kanarek, A., Lehmann, W., & Awkerman, J. (2018). A framework for linking population model development with ecological risk assessment objectives. *Integrated Environmental Assessment and Management*, 14(3), 369-380. https://doi.org/10.1002/ieam.2024

Raimondo, S., Schmolke, A., Pollesch, N., Accolla, C., Galic, N., Moore, A., Vaugeois, M., Rueda-Cediel, P., Kanarek, A., Awkerman, J. & Forbes, V. (2021). Pop-guide: Population modelling guidance, use, interpretation, and development for ecological risk assessment. *Integrated Environmental Assessment and Management,* 17(4), 767-784. <https://doi.org/10.1002/ieam.4377>

Werner, J., Palace, V. P., Wautier, K. G., Mills, K. H., Chalanchuk, S. M. & Kidd, K. A. (2006). Reproductive fitness of lake trout (*Salvelinus namaycush*) exposed to environmentally relevant concentrations of the potent estrogen ethynylestradiol (EE2) in a whole lake exposure experiment in Olivar, M. P. & Govoni, J. J. (Eds.) Recent advances in the study of fish eggs and larvae, *Scientia Marina* 70(Suppl.2): 5-6 (2006)
